# Synthesis of polyoxothiometalates through site-selective post-editing sulfurization of polyoxometalates†

**DOI:** 10.1039/d4sc02912g

**Published:** 2024-06-14

**Authors:** Kentaro Yonesato, Kazuya Yamaguchi, Kosuke Suzuki

**Affiliations:** a Department of Applied Chemistry, School of Engineering, The University of Tokyo 7-3-1 Hongo, Bunkyo-ku Tokyo 113-8656 Japan ksuzuki@appchem.t.u-tokyo.ac.jp k-yonesato@g.ecc.u-tokyo.ac.jp

## Abstract

Polyoxometalates (POMs) function as platforms for synthesizing structurally well-defined inorganic molecules with diverse structures, metals, compositions, and arrangements. Although post-editing of the oxygen sites of POMs has great potential for development of unprecedented structures, electronic states, properties, and applications, facile methods for site-selective substitution of the oxygen sites with other atoms remain limited. Herein, we report a direct site-selective oxygen–sulfur substitution method that enables transforming POMs [XW_12_O_40_]^4−^ (X = Si, Ge) to Keggin-type polyoxothiometalates (POTMs) [XW_12_O_28_S_12_]^4−^ using sulfurizing reagents in an organic solvent. The resulting POTMs retain the original Keggin-type structure, with all 12 surface W=O groups selectively converted to W=S without sulfurization of other oxygen sites. These POTMs show high stability against water and O_2_ in organic solvents and a drastic change in the electronic states and redox properties. The findings of this study represent a facile method for converting POMs to POTMs, leading to the development of their unique properties and applications in diverse fields, including (photo)catalysis, sensing, optics, electronics, energy conversion, and batteries.

## Abstract 2

This work presents a direct site-selective oxygen–sulfur substitution method that enables transforming polyoxometalates [XW_12_O_40_]^4−^ (X = Si, Ge) to Keggin-type polyoxothiometalates [XW_12_O_28_S_12_]^4−^ using sulfurizing reagents in an organic solvent.

## Graphical abstract

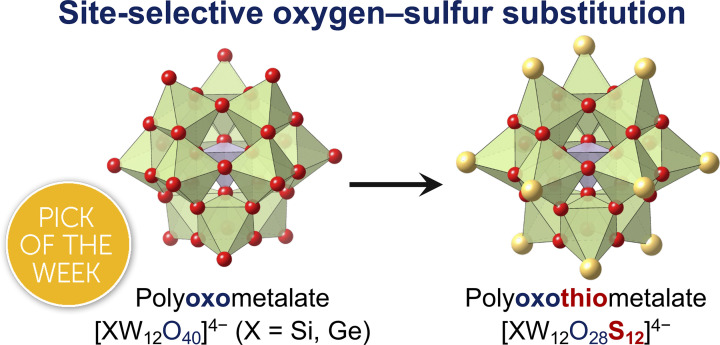

## Introduction

As a member of group 16, sulfur shares its elemental classification with that of oxygen. Nevertheless, metal sulfides exhibit distinct structures, electronic states, properties, (photo)catalysis, and applications that significantly differ from those of metal oxides because of the strong affinity of sulfur for metal atoms, unique redox properties, and narrow band gaps.^1^ Therefore, as seen in the photocatalysis of sulfur-doped titania^2^ and the hydrodesulfurization catalysis of molybdenum oxides activated by hydrogen sulfides,^3^ the modification of metal oxides with sulfur results in unique physicochemical properties.

Polyoxometalates (POMs), which are anionic metal oxide clusters (*e.g.*, W^6+^, Mo^6+^, V^5+^, Nb^5+^, and Ta^5+^), exhibit diverse structures and properties, including acidity/basicity, redox properties, and photochemical properties, depending on the structures, constituting atoms, and electronic states.^4^ These features enable diverse applications that include catalysis, medicine, materials science, sensor, electronics, and batteries. The substitution of the constituting atoms of POMs is an important approach to modify their properties and achieve novel functions: for example, the replacement of metals in POMs has been widely investigated through the direct metal substitution or metal introduction into the vacant sites of lacunary POMs.^5,6^ In addition, the replacement of the oxygen sites of POMs with various organic ligands, such as phosphonates, silicates, imidos, and pyridines, is also an important approach to synthesize functional materials.^4*j*,7^ However, the substitution of the oxygen sites with other atoms is still limited due to the difficulty in controlling the reactivity.^8^

In the field of synthetic organic chemistry, molecular post-editing has recently become increasingly important to realize late-stage chemical transformations.^9^ Considering the diverse structures of POMs, site-selective post-editing of POMs has great potential for the development of inorganic molecules with novel properties and applications. This study proposes a selective oxygen–sulfur substitution approach that enables facile and versatile synthesis of polyoxothiometalates (POTMs) from POMs. Several structurally defined POTMs have been synthesized by the self-condensation of mono-, di-, and trinuclear (oxo)thiometalates, or the reaction of these species with organic ligands and/or POMs.^10^ However, the direct oxygen–sulfur substitution reactions of POMs are limited to the oxygen sites on the substituted metal. For example, the mononiobium and monotantalum-oxo units in Lindqvist-type [(M^5+^O)W_5_O_18_]^3−^ and Keggin-type [(M^5+^O)PW_11_O_39_]^4−^ (M = Nb, Ta) have been converted to mono-sulfurized species [(M^5+^S)W_5_O_18_]^3−^ and [(M^5+^S)PW_11_O_39_]^4−^, respectively.^11^ These results showed that sulfurization proceeds only against terminal Nb^5+^=O and Ta^5+^=O, and not on tungstate, which critically hinders the investigation of POTMs. Therefore, the development of site-selective oxygen–sulfur substitution reactions for polyoxotungstates is crucial for establishing a facile and widely applicable method for exchanging the oxygen atoms of various POM precursors.

**Fig. 1:**
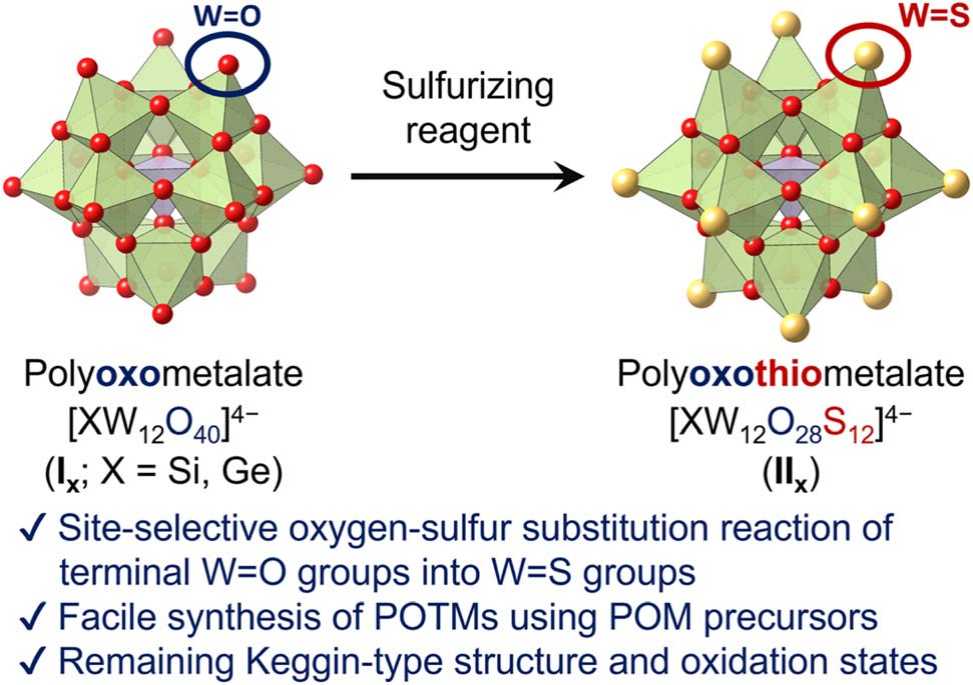
Schematic of the Keggin-type POTMs [XW_12_O_28_S_12_]^4−^ (X = Si, Ge) synthesized *via* site-selective sulfurization.

Herein, we report the first synthesis method of Keggin-type POTMs [XW_12_O_28_S_12_]^4−^ (II_X_; X = Si, Ge) through direct site-selective oxygen–sulfur substitution of the parent POMs [XW_12_O_40_]^4−^ (I_X_; Fig. 1). By reacting Keggin-type POMs and sulfurizing reagents in organic solvents, the 12 terminal (surface) W=O groups are selectively converted to W=S groups without undesirable structural changes or over-sulfurization. We also show that the resultant POTMs exhibit high stability and unique electronic states and redox properties, indicating that this sulfurizing method enables the post-editing sulfurization of POMs with various structures, constituent elements, electronic states, and physicochemical properties.

## Results and discussion

We first examined the oxygen atom substitution of Keggin-type silicotungstate ([SiW_12_O_40_]^4−^; I_Si_) to sulfur atoms using bis(trimethylsilyl)sulfide (TMS_2_S), which is a widely employed sulfurizing reagent for d^0^ metal–oxo species.^12^ However, the electrospray ionization (ESI)-mass spectrum showed that the oxygen atom substitution of I_Si_ did not proceed using TMS_2_S (12 equivalents with respect to I_Si_) in acetonitrile (Fig. S1a†). This result was consistent with that of previous reports showing that the reaction of Nb- or Ta-substituted polyoxotungstates and trialkylsilylsulfides converted the Nb=O and Ta=O groups into the Nb=S and Ta=S groups, respectively, while the other oxygen atoms remained intact.^11*a*,*c*^

Accordingly, we investigated the reactivity of several sulfurizing reagents toward POMs. When the tetra-*n*-butylammonium (TBA) salt of I_Si_ (TBA_4_[SiW_12_O_40_]) was reacted with Lawesson's reagent^13^ (three equivalents with respect to I_Si_) in acetonitrile at room temperature (∼25 °C), the reaction solution turned colorless to yellow (see ESI† for details). The positive-ion ESI-mass spectrum of the reaction solution revealed a series of signals with the *m*/*z* = 16 (*z* = 1) difference, indicating that the oxygen atoms of I_Si_ were substituted with sulfur atoms (Fig. S2a†).

**Fig. 2:**
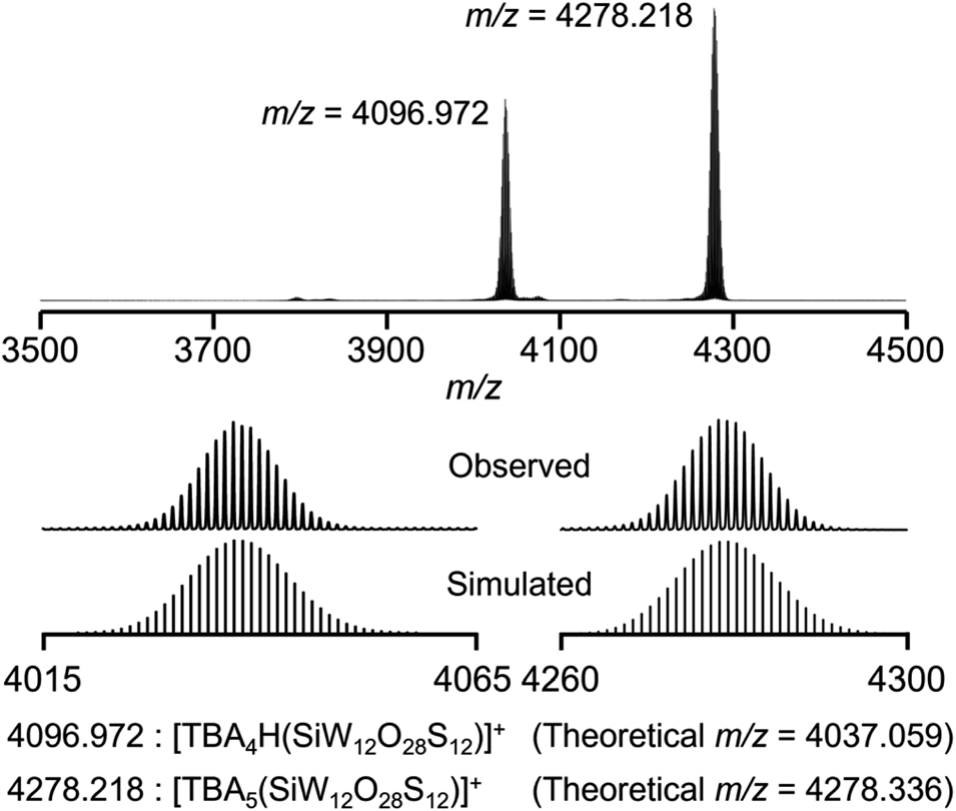
Positive-ion ESI-mass spectrum of II_Si_ in acetonitrile.

After further modification of the reaction conditions, the use of six equivalents of Lawesson's reagent provided an ESI-mass spectrum with two prominent signal sets at *m*/*z* = 4036.972 and 4278.218 (Fig. 2 and S2†). These signal sets were assigned to [TBA_4_H(SiW_12_O_28_S_12_)]^+^ (theoretical *m*/*z* = 4037.059) and [TBA_5_(SiW_12_O_28_S_12_)]^+^ (theoretical *m*/*z* = 4278.336), showing that 12 out of 40 oxygen atoms were substituted with sulfur atoms to form [SiW_12_O_28_S_12_]^4−^ (II_Si_). In addition, even after the reaction of I_Si_ with excess amounts of Lawesson's reagent (9, 12, and 20 equivalents with respect to I_Si_), the ESI-mass spectra showed that sulfur atoms were not further introduced into I_Si_, showing that Lawesson's reagent can selectively convert I_Si_ to II_Si_ (Fig. S2c–e†). Based on the above results and elemental analysis, the formula of II_Si_ was determined as TBA_4_[SiW_12_O_28_S_12_]. Note that the ESI-mass spectrum of II_Si_ in acetonitrile containing water (*ca.* 2000 equivalents with respect to II_Si_) under air showed no significant changes, revealing the high stability of II_Si_ against water and O_2_ (Fig. S3†). When diphosphorus pentasulfide was used as a sulfuring reagent, I_Si_ was not completely converted to II_Si_, and several terminal oxygen atoms remained likely due to the very low solubility of diphosphorus pentasulfide in acetonitrile (Fig. S1b†). In contrast, although triphenylphosphine sulfide and dimethyl trisulfide exhibited good solubility, they did not react with I_Si_ under the same conditions (Fig. S1c and d†).

**Fig. 3:**
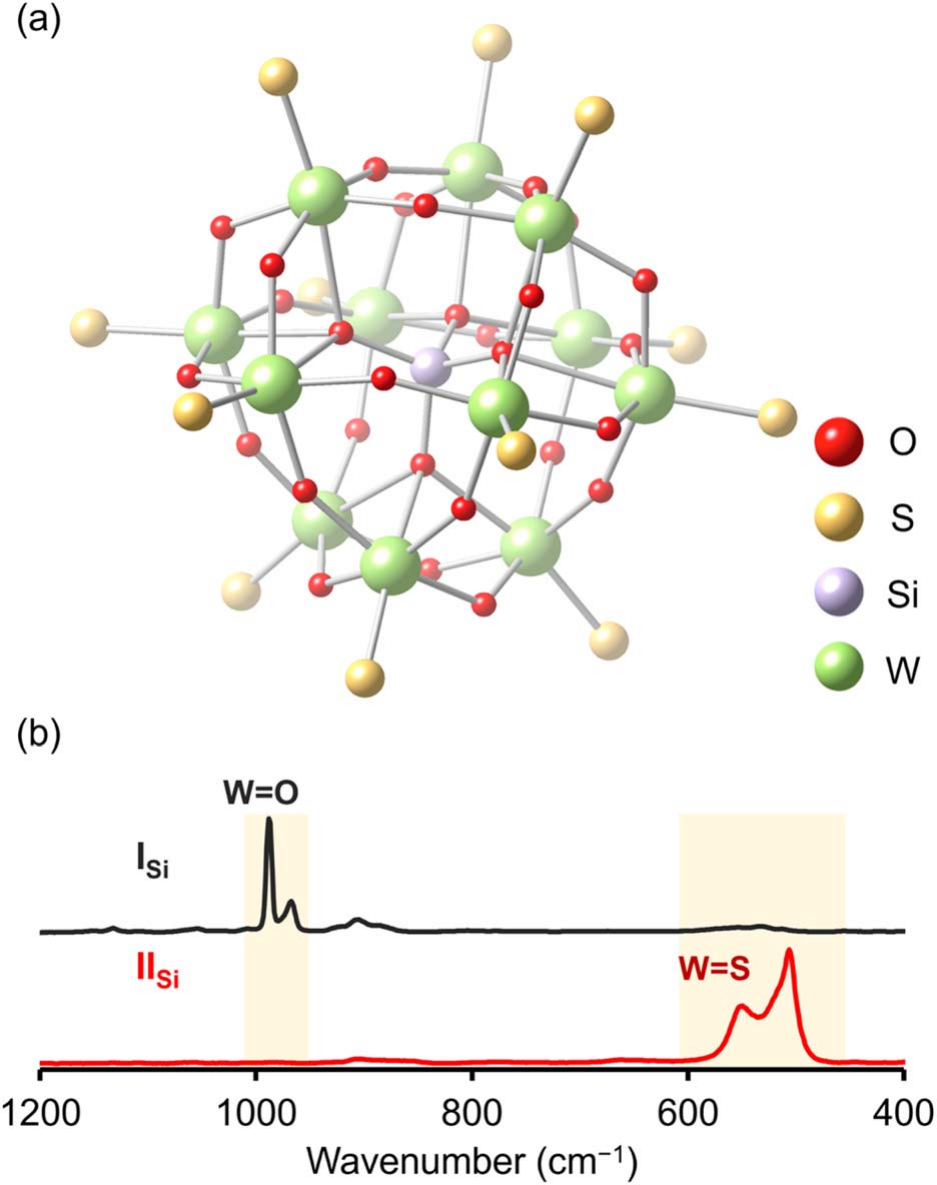
(a) Ball-and-stick representation of the crystal structure of the anion part of II_Si_. (b) Raman spectra of I_Si_ (black line) and II_Si_ (red line).

Since X-ray crystallographic analysis of the TBA salt of II_Si_ was unsuccessful likely because of the flexibility of TBA cations, crystallographic analysis was performed using the tetraphenylphosphonium (TPP) salt, which was obtained *via* cation exchange reaction of II_Si_ with TPPBr (see ESI† for detail). The elemental analysis revealed that the formula of the TPP salt was TPP_4_[SiW_12_O_28_S_12_], showing that 12 sulfur atoms were retained and all TBA cations were exchanged with TPP cations. X-ray crystallographic analysis of the TPP salt of II_Si_ revealed that the α-Keggin-type {SiW_12_} structure was retained, and all 12 terminal oxygen atoms of I_Si_ (*i.e.*, the W=O groups in [SiW_12_O_40_]^4−^) were substituted with sulfur atoms (Fig. 3a, S4 and Table S1†). Notably, 28 other oxygen atoms remained, that is, four μ_4_-oxo atoms surrounding the heteroatom (Si) and 24 μ_2_-oxo atoms bridging the polyatoms (W). These results were consistent with the aforementioned ESI-mass analysis, showing that 12 out of 40 oxygen atoms were substituted with sulfur atoms upon reaction with Lawesson's reagent (Fig. 2). The W=S bond lengths in II_Si_ ranged from 2.11 to 2.17 Å, clearly longer than the terminal W=O bonds of I_Si_ (1.63–1.74 Å).^14^ The bond valence sum (BVS) values of the sulfur atoms ranged from 1.80 to 2.13 (Table S2†), indicating that each sulfur atom formed a double bond with a tungsten atom (W=S group). The BVS values of the Si (3.89, 3.90) and W (6.12–6.61) atoms also showed that their oxidation states remained at +4 and +6, respectively (Table S3†).

The Raman spectrum of I_Si_ showed prominent peaks corresponding to the stretching vibrations of the W=O bonds (967 and 988 cm^−1^; Fig. 3b).^15^ In contrast, the Raman spectrum of II_Si_ depicted no stretching vibrations of the W=O bonds, but clearly illustrated those corresponding to the W=S bonds in the 500–600 cm^−1^ region (Fig. 3b).^16^ The FT-IR spectrum of II_Si_ also showed the sharp peak at 493 cm^−1^ assignable to the stretching vibration of W=S bonds (Fig. S5†). These results supported the successful synthesis of the Keggin-type POTM [SiW_12_O_28_S_12_]^4−^ (II_Si_) *via* the oxygen–sulfur substitution reactions of W=O into W=S. This is the first report on the synthesis of a structurally defined POTM, in which all the terminal W=O groups of parent POM were converted to W=S. Previously reported Keggin-type POTMs [γ-XW_10_O_36_(M^5+^_2_S_2_O_2_)]^*n*−^ (X = Si, P; M = W, Mo) were synthesized by introducing the [M^5+^_2_S_2_O_2_]^2+^ moiety into the vacant sites of lacunary POMs [XW_10_O_36_]^*n*−^, wherein two S atoms bridged two M^5+^ atoms of the [M^5+^_2_S_2_O_2_]^2+^ moiety (*i.e.*, M^5+^–S^2−^–M^5+^).^11*b*,*c*^ In contrast, we demonstrated that the direct oxygen–sulfur substitution reaction of POMs enabled the selective incorporation of sulfur atoms at terminal (surface) sites (*i.e.*, W^6+^ = S).

**Fig. 4:**
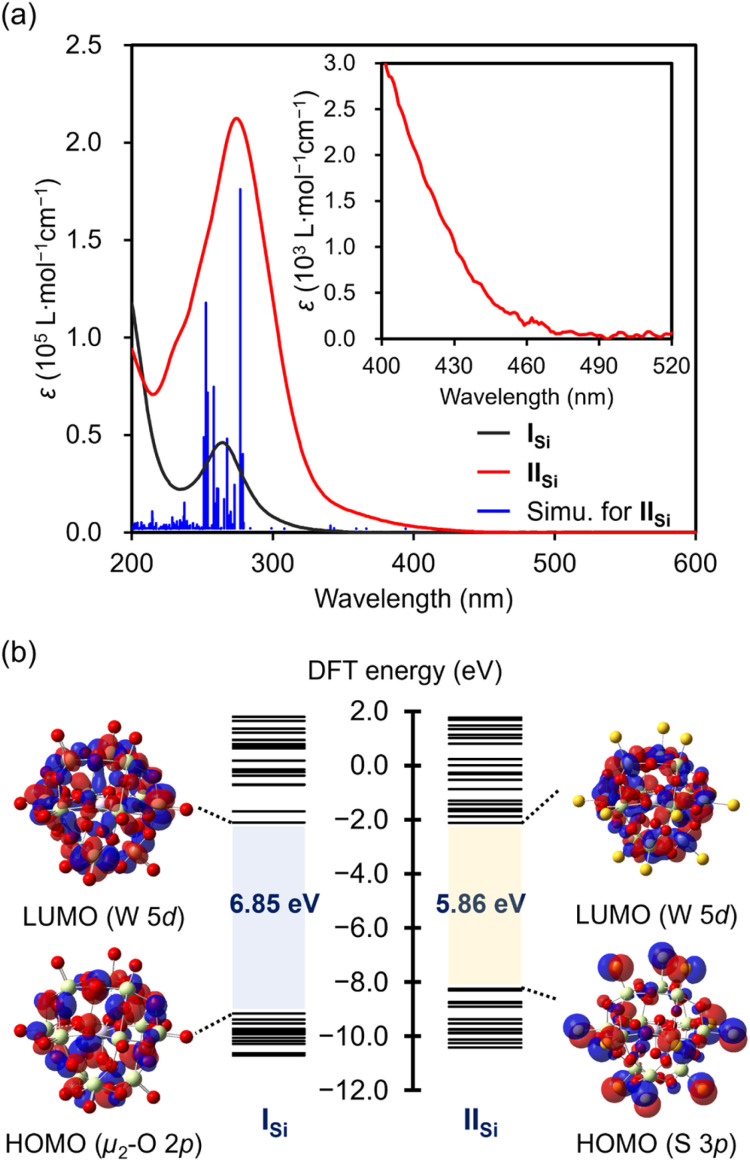
(a) UV-vis spectra of I_Si_ (black line) and II_Si_ (red line), and simulation of II_Si_ based on the TD-DFT study (blue bar). (b) Energy diagrams and visualization of the HOMO and the LUMO of I_Si_ and II_Si_.

The UV-vis spectrum of II_Si_ in acetonitrile exhibited a prominent absorption band at *λ* = 274 nm (*ε* = 2.1 × 10^5^ L mol^−1^ cm^−1^), which was observed with a higher intensity at a lower wavelength compared with that of I_Si_ (*λ* = 264 nm; *ε* = 4.6 × 10^4^ L mol^−1^ cm^−1^) (Fig. 4a). The acetonitrile solution of I_Si_ was colorless and showed no absorption band in the visible light region, whereas that of II_Si_ was pale yellow and exhibited weak absorption bands up to approximately 470 nm (Fig. S6†). These results indicated that the introduction of the sulfur atoms led to a drastic change in the electronic state. Thus, to investigate the electronic state, we conducted density functional theory (DFT) calculations on I_Si_ and II_Si_. In the case of I_Si_, the highest occupied molecular orbital (HOMO) was mainly derived from the bridging μ_2_-oxo atoms (Fig. 4b and S7†). In contrast, with the introduced sulfur atoms, the occupied orbitals of II_Si_ (HOMO–HOMO−12) were mainly derived from the terminal S 3p orbitals. The HOMO–LUMO energy gap of II_Si_ became smaller than I_Si_ (6.85 eV for I_Si_ and 5.86 eV for II_Si_, Fig. 4b and S8†). The lowest unoccupied molecular orbitals (LUMOs) of I_Si_ and II_Si_ were mainly derived from W 5d orbitals. Based on the time-dependent (TD) DFT study, the absorption bands of I_Si_ were assigned to the ligand-to-metal charge transfer from the oxygen atoms to the tungsten atoms. Meanwhile, the absorption bands of II_Si_ in the UV region (*λ*_max_ = 274 nm) were mainly attributed to the excitation from the S 3p orbitals (HOMO–HOMO−12) and the W–S bonding orbitals (HOMO−13–HOMO−19) to the W–S antibonding orbitals (LUMO+2–LUMO+5). In addition, the broad absorption bands at the longer wavelengths (*λ* > 350 nm) were mainly attributed to the excitation from the S 3p orbitals and the W–S bonding orbitals to the W 5d orbitals (LUMO, LUMO+1, LUMO+6–LUMO+8) (Fig. S9†). These results revealed the significant contribution of sulfur atoms in the optical properties of II_Si_.

We evaluated the redox behavior of II_Si_ using cyclic voltammetry in acetonitrile. POTM II_Si_ exhibited stable redox cycles, indicating high stability during the reduction/reoxidation reactions (Fig. S10†). Two reduction waves of II_Si_ were observed at −1.08 and −1.48 V (*vs.* Ag/Ag^+^ reference electrode), showing that the first redox potential of II_Si_ was similar to that of I_Si_, whereas the second redox potential shifted from that of I_Si_ (−1.62 V) after the oxygen atom substitution to sulfur atoms.

Finally, in addition to the sulfurization of Si-centered Keggin-type POM (I_Si_), we investigated the site-selective sulfurization of Ge-centered [GeW_12_O_40_]^4−^ (I_Ge_) using Lawesson's reagent. ESI-mass spectrum revealed that site-selective sulfurization of I_Ge_ also proceeded to form [GeW_12_O_28_S_12_]^4−^ (II_Ge_) (Fig. S11†). These results suggest that this method is potentially applicable to sulfurization of various heteropolyoxotungstates.

## Conclusions

We have developed a novel synthetic method for polyoxothiotungstates through direct site-selective substitution of terminal oxo ligands (W=O groups) of polyoxotungstates into sulfide ligands (W=S groups) *via* reaction with sulfurizing reagents (*e.g.*, Lawesson's reagent). The resulting POTMs (*i.e.*, II_X_, X = Si, Ge) retained the parent Keggin-type structure (*i.e.*, I_X_), while their electronic states and redox properties drastically changed from those of I_X_. This finding holds the potential to pave the way for the development of the sulfurization method for a broad range of POMs, allowing atomically precise design of molecular metal oxides and sulfides with diverse structures, constituent elements, compositions, and properties. We believe that such advancements will lead to the diverse applications of POMs and related materials, including (photo)catalysis, sensing, optics, energy conversion, and batteries.

## Data availability

The data supporting this manuscript is available in the ESI† of and available on request. Crystallographic data for a TPP salt of II_Si_ has been deposited at the CCDC (deposition number 2322226).

## Author contributions

K. Yo. and K. S. design the project and experiments. K. Yo. performed the major parts of experiments. K. Yo, K. S., and K. Ya cowrote the manuscript.

## Conflicts of interest

There are no conflicts to declare.

## Supplementary Material

SC-015-D4SC02912G-s001

SC-015-D4SC02912G-s002
