# Supplementary material for: Synthesis of polyoxothiometalates through site-selective post-editing sulfurization of polyoxometalates
Source: Chem Sci. 2024 Jun 14;15(29):11267–71. doi: 10.1039/d4sc02912g (PMC11268463; doi:10.1039/d4sc02912g)
Supplement: SC-015-D4SC02912G-s001 [file SC-015-D4SC02912G-s001.pdf]

Electronic Supplementary Material (ESI) for Chemical Science.
This journal is © The Royal Society of Chemistry 2024

Electronic Supplementary Information (ESI) for

Synthesis of polyoxothiometalates through site-selective post-editing sulfurization of polyoxometalates

Kentaro Yonesato,*[a] Kazuya Yamaguchi and Kosuke Suzuki*[a]

[a] Department of Applied Chemistry, School of Engineering, The University of Tokyo, 7-3-1 Hongo, Bunkyo-ku, Tokyo 113-8656, Japan.

E-mail: ksuzuki@appchem.t.u-tokyo.ac.jp; k-yonesato@g.ecc.u-tokyo.ac.jp

Contents **Page**

Contents	Page
Experimental section	S2–S3
Table S1–S3	S4
Figs. S1–S11	S5–S12
References	S13

Experimental Section

Materials

Acetonitrile, dichloromethane, and diethyl ether were purchased from Kanto Chemical. The solvents were dried and stored with appropriate molecular sieves. Lawesson's reagent (2,4-bis(4-methoxyphenyl)-1,3,2,4-dithiadiphosphetane-2,4-disulfide), bis(trimethylsilyl)sulfide, triphenylphosphine sulfide, dimethyl trisulfide, tetra-*n*-butylammonium (TBA) bromide (TBABr), tetraphenylphosphonium (TPP) bromide (TPPBr) were obtained from Tokyo Chemical Industry. Diphosphorus pentasulfide was purchased from Merck Sigma-Aldrich. $H_4SiW_{12}O_{40}$ was purchased from FUJIFILM WAKO Chemicals. $TBA_4SiW_{12}O_{40}$ was synthesized by the reaction of $H_4SiW_{12}O_{40}$ and TBABr according to the reported procedure.[S1]

Instrument

Elemental analyses for carbon, hydrogen, nitrogen, and sulfur were performed on a MICRO CORDER JM10, HSU-20, and ICS-1100 at the Open Facility Center, Tokyo Institute of Technology. Ultraviolet–visible absorption spectra were measured using a Shimadzu UV-3600 Plus with a 1 cm quartz cell at room temperature (~25 °C). IR spectra were measured on a JASCO FT/IR-4100 spectrometer using KBr disks. Raman spectra were measured on a JASCO NRS-5100 spectrometer under an irradiation laser of wavelength λ = 532 nm (0.1 mW). ESI-mass spectra were recorded on a Shimadzu LCMS-9050 instrument and a Waters Xevo G2-XS QTof instrument. Cyclic voltammetry measurements were performed with a BioLogic VSP-300 at the scan rate of 50 mV s^{-1}. A standard three-electrode arrangement was employed with a glassy carbon disk electrode as the working electrode and a platinum wire as the counter electrode. The potentials were measured using $Ag/AgNO_3$ reference electrode (10 mM $AgNO_3$, 100 mM $TBAClO_4$ in acetonitrile).

Synthesis of $TBA_4[SiW_{12}O_{28}S_{12}]$ (II_{Si}): To a 30 mL of acetonitrile solution of $TBA_4SiW_{12}O_{40}$ (**I_{Si}**; 1.20 g, 0.31 mmol), Lawesson's reagent (1.00 g, 2.47 mmol) was added, and the solution was stirred for a day at room temperature (~25 ºC). After filtration, the resultant solution was added dropwise to excess diethyl ether (600 mL). The yellow precipitate formed was collected by filtration. The yellow powder of **II_{Si}** was obtained after washing with dichloromethane (1.07 g, 85% yield). **II_{Si}** was further purified by recrystallization from a mixture of acetonitrile and diethyl ether (~55% yields, yellow needle crystals). Positive-ion MS (ESI, acetonitrile): *m/z* 4036.972 (calcd. 4037.059 for $[TBA_4H(SiW_{12}O_{28}S_{12})]^+$), 4278.218 (calcd. 4028.336 for $[TBA_5(SiW_{12}O_{28}S_{12})]^+$). IR (KBr pellet, cm^{-1}) 379, 493, 520, 779, 868, 943, 1378, 1479, 1629, 2872, 2930, 2959, 3435. Elemental analysis calcd (%) for $TBA_4[SiW_{12}O_{28}S_{12}]$: C, 19.04; H, 3.60; N, 1.39; S, 9.53. Found: C, 18.94; H, 3.47; N, 1.43; S, 9.41. UV-Vis (acetonitrile solution): λ (ε) 274 nm (2.1×10^5 $M^{-1}cm^{-1}$).

Synthesis of $TPP_4[SiW_{12}O_{28}S_{12}]\cdot(CH_3CN)_2$ (TPP salt of II_{Si}): The crystallographic analysis was conducted for the tetraphenylphosphonium (TPP) salt. To an acetonitrile solution (1 mL) of a TBA salt of **II_{Si}**, an acetonitrile solution containing 4 equivalents of TPPBr (100 µL) was added quietly. After standing the solution overnight, the TPP salt was obtained as yellow plate single crystals suitable for crystallographic analysis. Elemental analysis calcd (%) for $TPP_4[SiW_{12}O_{28}S_{12}]\cdot(CH_3CN)_2$: C, 26.65; H, 1.92; N, 0.62; S, 8.54. Found: C, 26.60; H, 2.17; N, 0.77; S, 8.40.

X-ray crystallographic analysis

Single-crystal X-ray diffraction measurements were made on BL02B1 beamline at the SPring-8 facility of the Japan Synchrotron Radiation Research Institute (Proposal number; 2023A1731, 2023B1842) with a PILATUS3 X CdTe 1M detector at −173°C. The incident X-ray beam (λ = 0.4132 Å) was monochromatized by a Si(311) double-crystal monochromator. The data collection and process were conducted using RAPID AUTO and CrysAlisPro software[S2], respectively. In the reduction of data, Lorentz, polarization, and empirical absorption corrections were made. The structural analyses were performed using Olex[2] and WinGX.[S3,S4] Structures were solved using SHELXT-2018/1 (intrinsic phasing methods) and refined by SHELXL-2018/3.[S5,S6] All non-hydrogen atoms (C, O, Si, P, S, and W) were refined anisotropically. CCDC-2322226 contains the supplementary crystallographic data. The data can be obtained free of charge from The Cambridge Crystallographic Data Centre via www.ccdc.cam.ac.uk/data_request/cif.

Bond valence sum (BVS) calculations

The BVS values were calculated by the expression for the variation of the length r_{ij} of a bond between two atoms *i* and *j* in observed crystal with valence V*i*.

$$V_i = \sum \exp\left(\frac{r'_0 - r_{ij}}{B}\right)$$

where *B* is constant equal to 0.37 Å, r'_0 is bond valence parameter for a given atom pair.[S7]

DFT calculations

DFT calculations were performed using Gaussian 16, Rev. B.01. The geometries used in the calculation were based on the crystal structures determined in this study. The anion structures of $\mathbf{I_{Si}}$ and $\mathbf{II_{Si}}$ were optimized at the CAM-B3LYP functional with 6-31G(d) for Si, O and S, and LanL2DZ for W by using the polarizable continuum model with the parameters of the integral equation formalism model for acetonitrile.

Table S1. Crystallographic parameters of the TPP salt of $\mathbf{II}_{\mathrm{Si}}$.

Formula	$C_{100}H_{86}N_2P_4SiW_{12}O_{28}S_{12}$
Crystal system	Triclinic
Space group	*P*1
a (Å)	13.6691(2)
b (Å)	14.5906(2)
c (Å)	17.0239(2)
α (deg)	67.9371(13)
β (deg)	68.7098(13)
γ (deg)	89.8358(11)
V (Å^3)	2896.62(8)
Z	1
Temperature (K)	100(2)
ρ_{calc} (g cm^{-3})	2.560
R_1 [$I > 2\sigma(I)$]	0.0245
wR_2	0.0684
GOF	1.040

Table S2. BVS values for S atoms of the TPP salt of $\mathbf{II}_{\mathrm{Si}}$.

S1A	1.93	S2A	1.95	S3A	1.88	S4A	1.86
S5A	1.98	S6A	1.82	S7A	1.95	S8A	1.99
S9A	1.86	S10A	1.92	S11A	1.83	S12A	1.91
S1B	2.01	S2B	1.86	S3B	1.80	S4B	2.08
S5B	1.91	S6B	1.98	S7B	1.86	S8B	1.83
S9B	1.85	S10B	2.06	S11B	2.02	S12B	2.13

Table S3. BVS values for Si and W atoms of the TPP salt of $\mathbf{II}_{\mathrm{Si}}$.

Si1	3.89	(for O1A-O4A)					
Si1	3.90	(for O1B-O4B)					
W1A	6.24	W2A	6.30	W3A	6.28	W4A	6.29
W5A	6.33	W6A	6.35	W7A	6.33	W8A	6.32
W9A	6.32	W10A	6.33	W11A	6.18	W12A	6.33
W1B	6.36	W2B	6.31	W3B	6.26	W4B	6.51
W5B	6.20	W6B	6.44	W7B	6.46	W8B	6.12
W9B	6.30	W10B	6.46	W11B	6.25	W12B	6.61

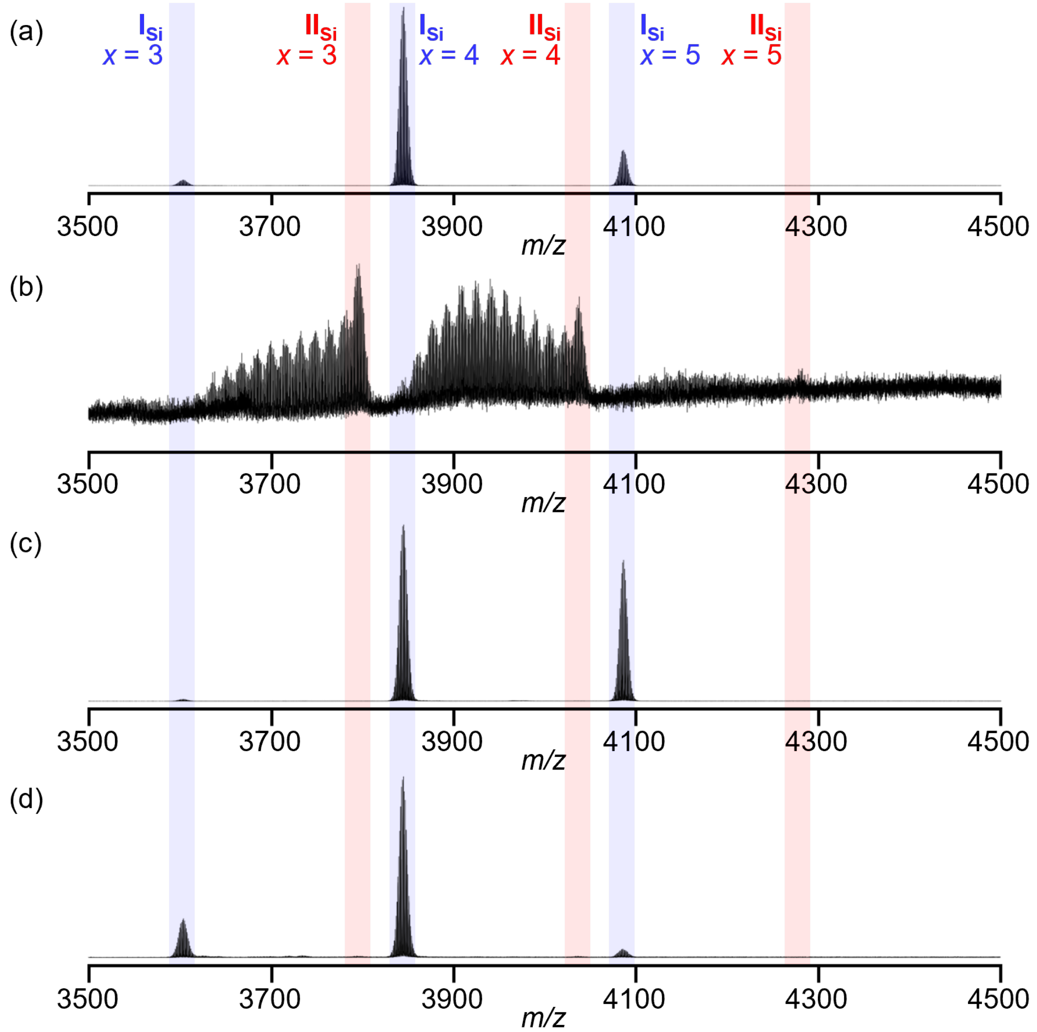

Fig. S1 Positive-ion ESI-mass spectra of $\mathbf{I_{Si}}$ after the reaction with various sulfurizing reagents in acetonitrile for 18 h at room temperature (~25 ºC): (a) bis(trimethylsilyl)sulfide (12 eq. with respect to $\mathbf{I_{Si}}$), (b) diphosphorus pentasulfide (6 eq.), (c) triphenylphosphine sulfide (12 eq.), and (d) dimethyl trisulfide (12 eq.). Blue and red highlights show the regions for the sets of signals assignable to $\mathbf{I_{Si}}$ (i.e., $[TBA_xH_{5-x}(SiW_{12}O_{40})]^+$; x = 3, 4, and 5) and $\mathbf{II_{Si}}$ (i.e., $[TBA_xH_{5-x}(SiW_{12}O_{28}S_{12})]^+$; x = 3, 4, and 5), respectively.

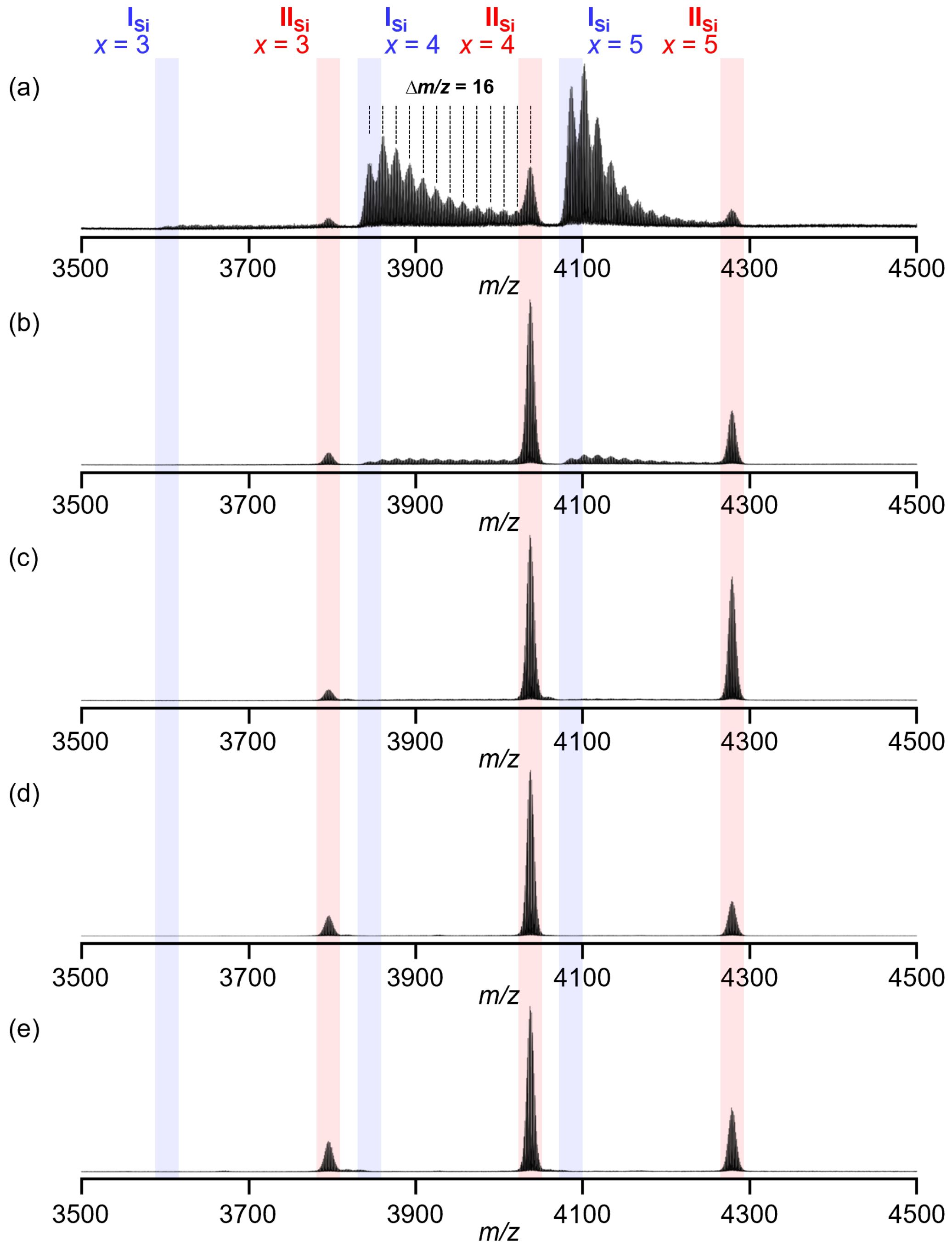

Fig. S2 Positive-ion ESI-mass spectra of $\mathbf{I_{Si}}$ after the reaction with various amounts of Lawesson's reagent in acetonitrile for 18 h at room temperature (~25 ºC): (a) 3 eq. of Lawesson's reagent with respect to $\mathbf{I_{Si}}$, (b) 6 eq., (c) 9 eq., (d) 12 eq., (e) 20 eq. Blue and red highlights show the regions for the sets of signals assignable to $\mathbf{I_{Si}}$ (i.e., $[TBA_xH_{5-x}(SiW_{12}O_{40})]^+$; x = 3, 4, and 5) and $\mathbf{II_{Si}}$ (i.e., $[TBA_xH_{5-x}(SiW_{12}O_{28}S_{12})]^+$; x = 3, 4, and 5), respectively.

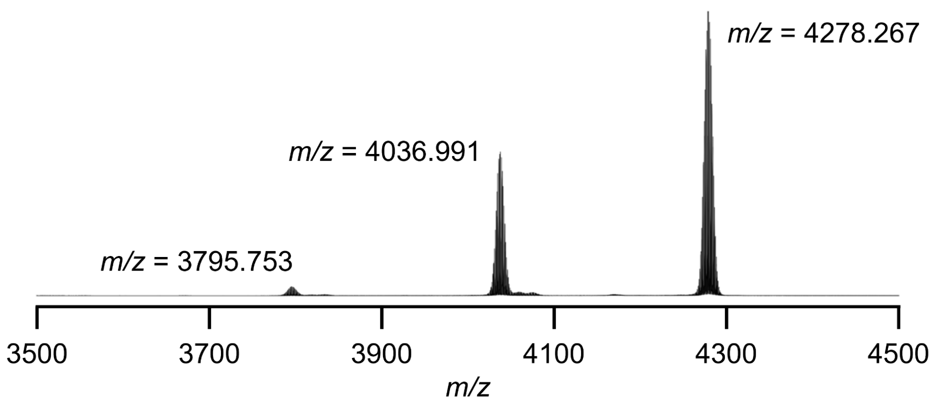

Fig. S3 Positive-ion ESI-mass spectrum of $\mathbf{II}_{Si}$ in acetonitrile containing water (1 vol%, ca. 2000 eq. with respect to $\mathbf{II}_{Si}$) after standing for 18 h. Three sets of signals centered at *m/z* = 3795.753, 4036.991 and 4278.267 are assignable to $[TBA_3H_2(SiW_{12}O_{28}S_{12})]^+$ (theoretical *m/z* = 3795.782), $[TBA_4H(SiW_{12}O_{28}S_{12})]^+$ (theoretical *m/z* = 4037.059) and $[TBA_5(SiW_{12}O_{28}S_{12})]^+$ (theoretical *m/z* = 4278.336), respectively.

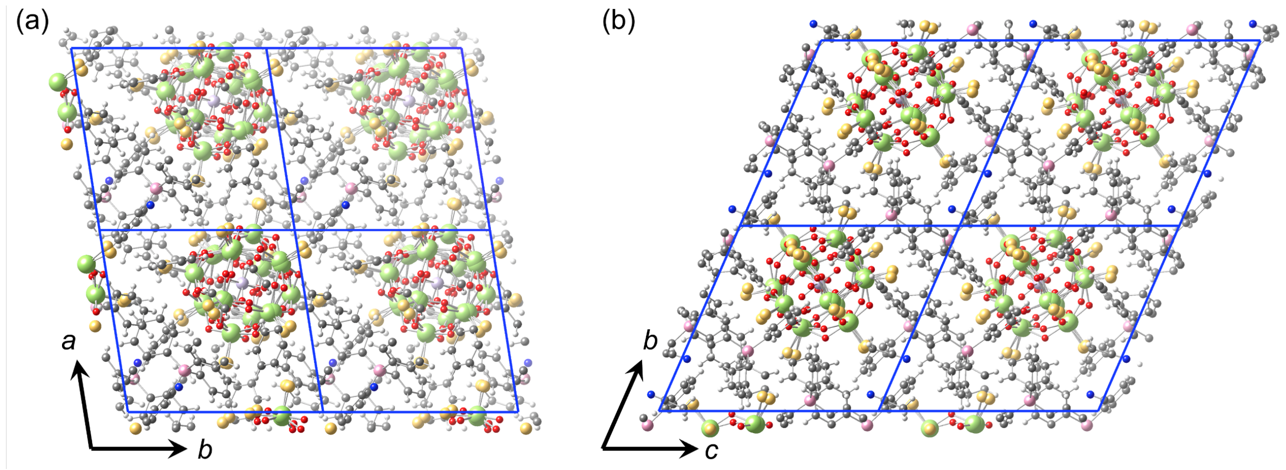

Fig. S4 Crystal packing of the TPP salt of $\mathbf{II}_{Si}$ viewing from (a) *c*-axis direction and (b) *a*-axis direction.

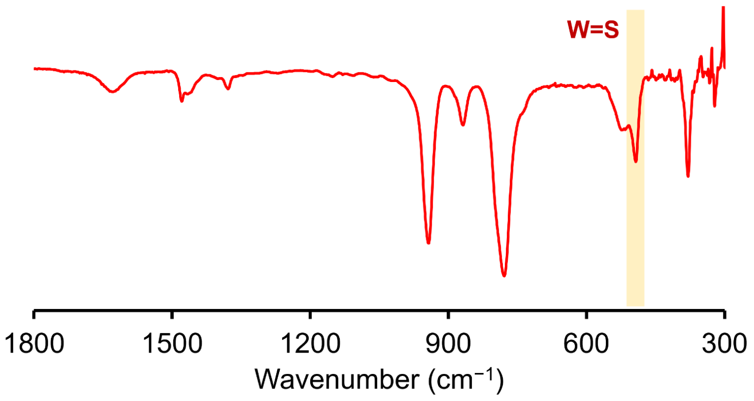

Fig. S5 FT-IR spectrum of $\mathbf{II_{Si}}$ (KBr pellet).

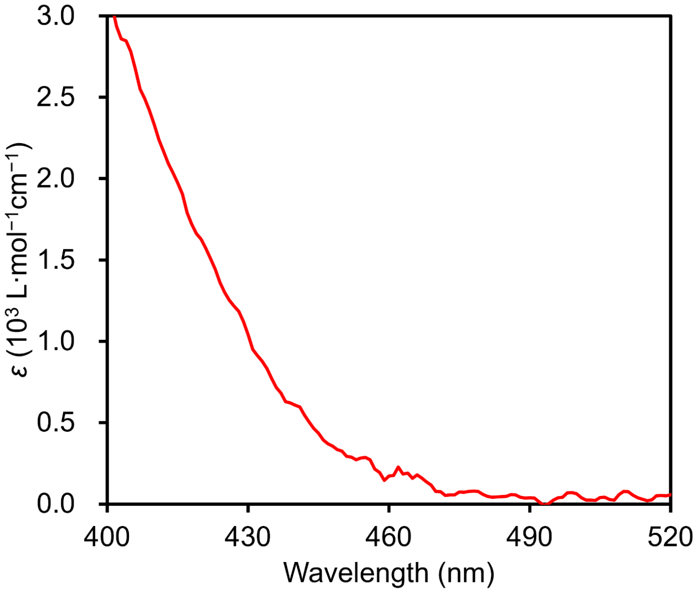

Fig. S6 Enlarged view of UV-vis spectrum of $\mathbf{II_{Si}}$ in acetonitrile around absorption-edge (ca. 470 nm).

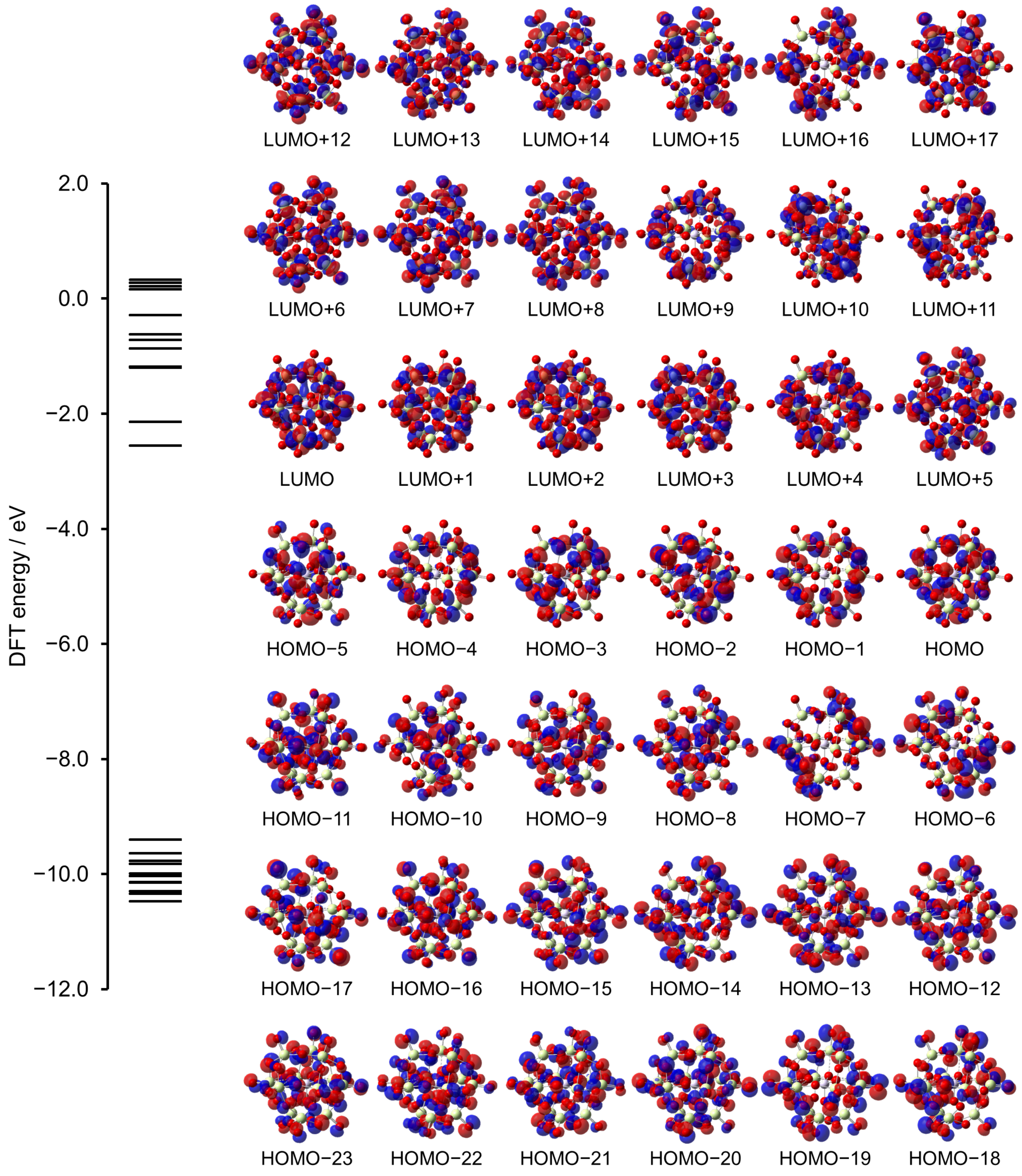

Fig. S7 Energy diagram and molecular orbitals of $\mathbf{I}_{\mathbf{Si}}$ based on the DFT study.

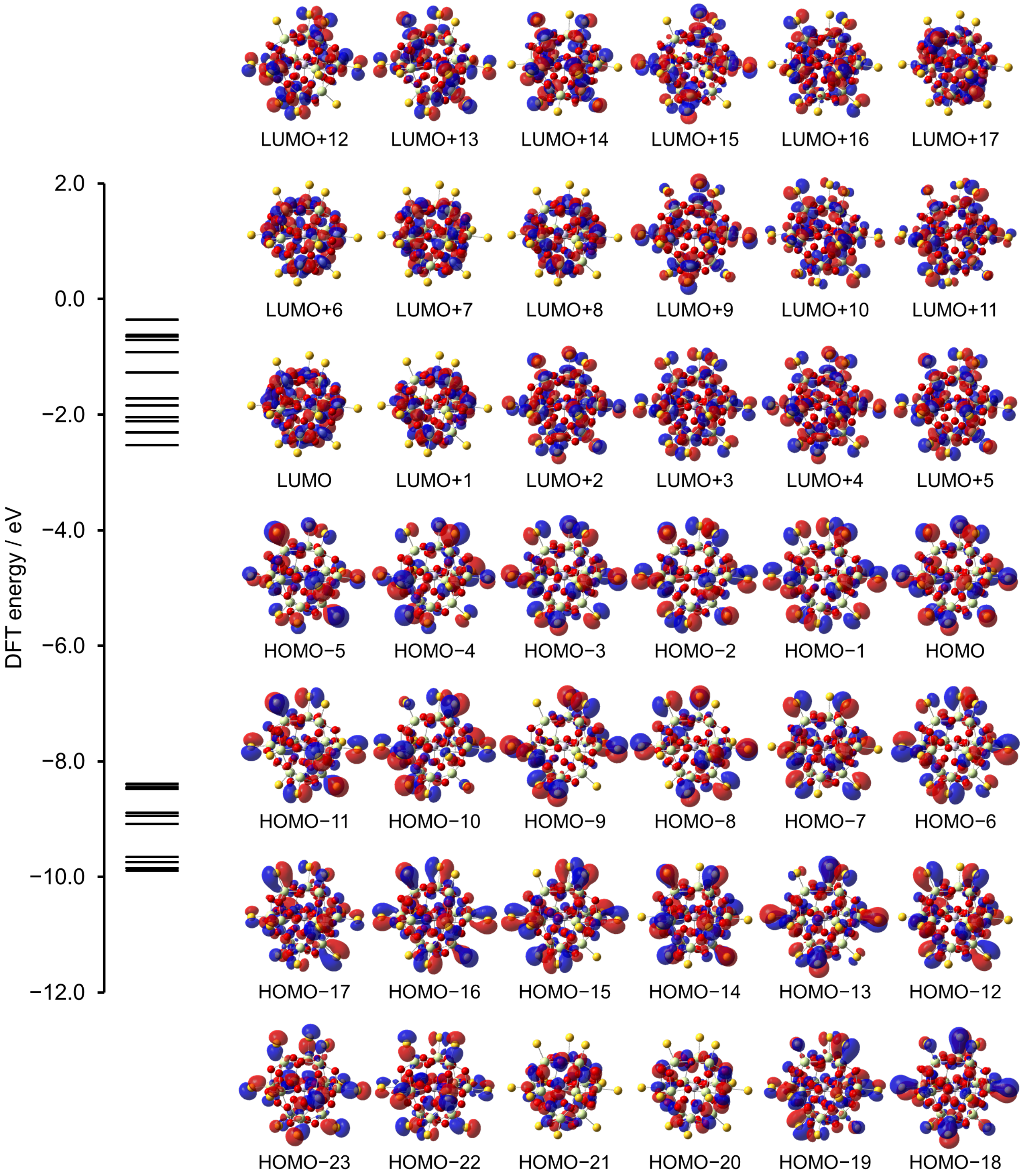

Fig. S8 Energy diagram and molecular orbitals of $\mathbf{II_{Si}}$ based on the DFT study.

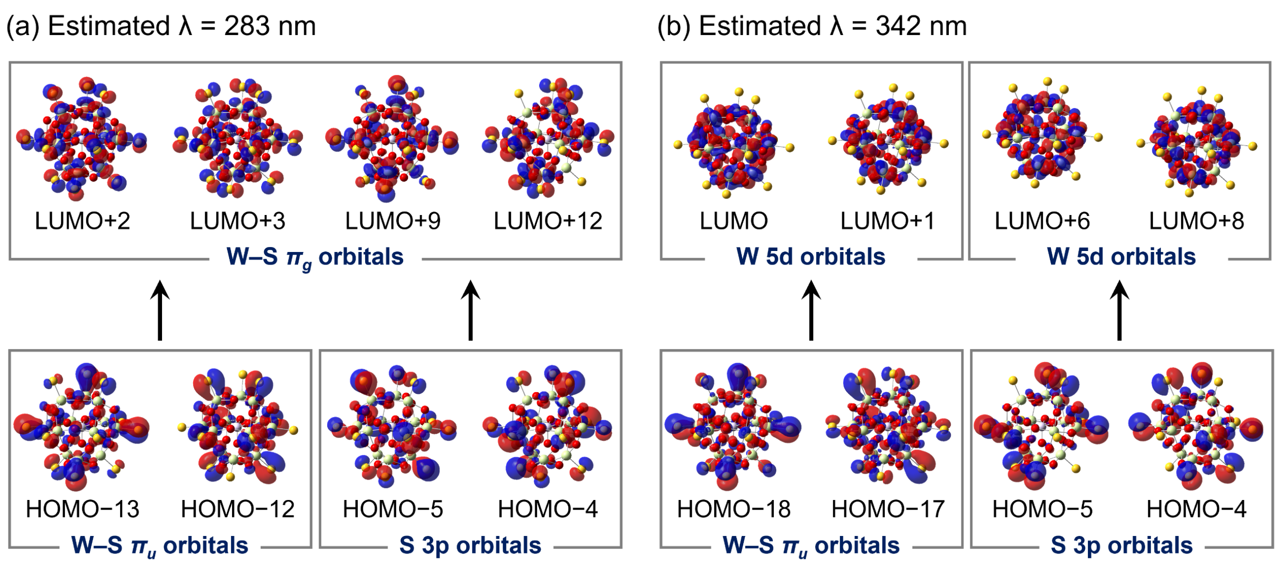

Fig. S9 Summary of the result of time-dependent DFT (TD-DFT) study on **II**$_{\mathbf{Si}}$ with the estimated absorption wavelength of (a) λ = 283 nm and (b) λ = 342 nm.

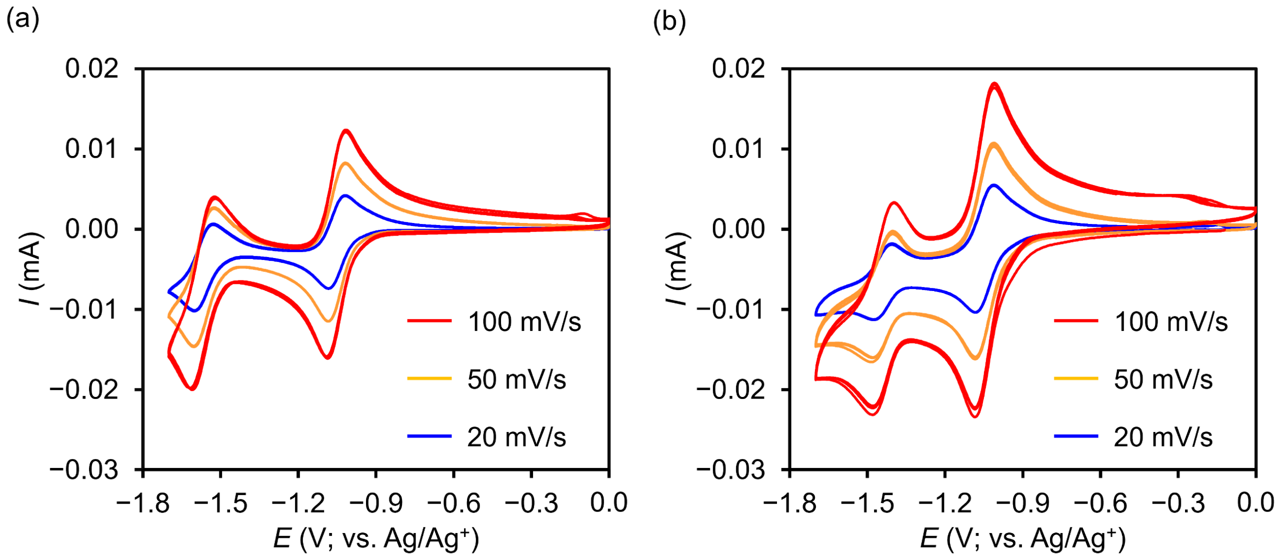

Fig. S10 Cyclic voltammograms of (a) **I**$_{\mathbf{Si}}$ and (b) **II**$_{\mathbf{Si}}$ in acetonitrile (10 μM, TBAClO$_4$ 0.10 mM).

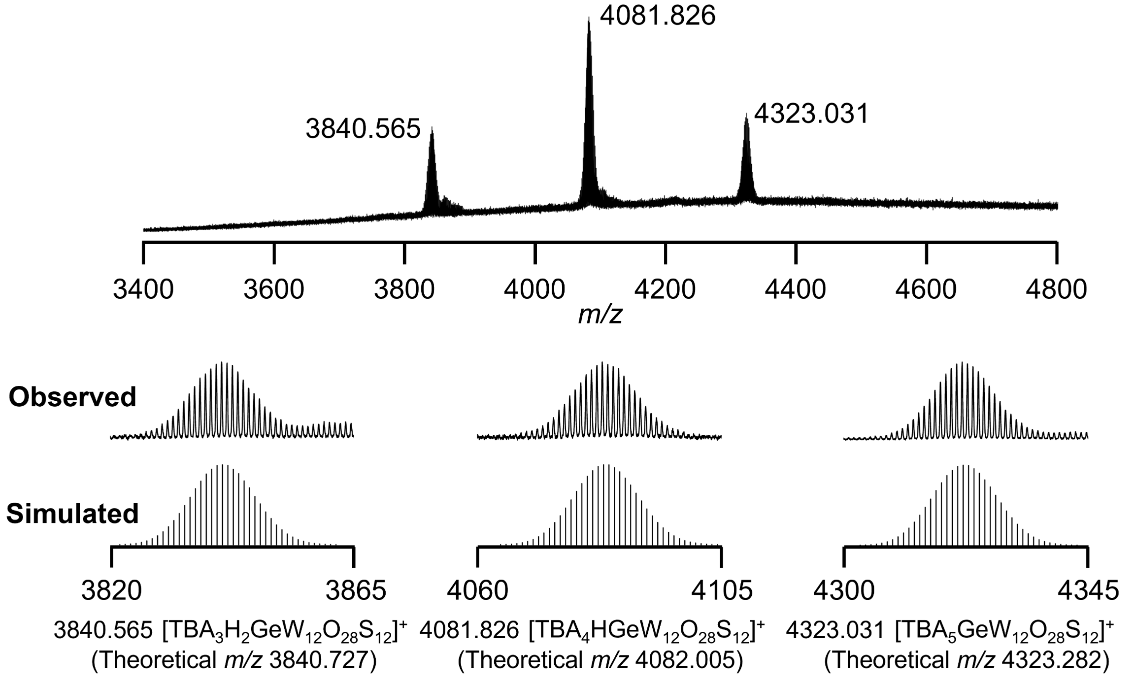

Fig. S11 Positive-ion ESI-mass spectrum of $\mathbf{I_{Ge}}$ after the reaction with Lawesson's reagent (9 eq. with respect to $\mathbf{I_{Ge}}$) in acetonitrile for 18 h at room temperature (i.e., $\mathbf{II_{Ge}}$).

References

S1. C. Rocchiccioli-Deltcheff, M. Fournier, R. Franck and R. Thouvenot, *Inorg. Chem.* **1983**, *22*, 207.

S2. Rigaku OD. CrysAlis PRO. Rigaku Oxford Diffraction Ltd, Yarnton, England (2018).

S3. O. V. Dolomanov, L. J. Bourhis, R. J. Gildea, J. A. K. Howard and H. Puschmann, *J. Appl. Cryst.* 2009, **42**, 339.

S4. L. J. Farrugia, *J. Appl. Cryst.* 2012, **45**, 849.

S5. G. M. Sheldrick, *Acta Crystallogr. A* 2015, **71**, 3.

S6. (a) G. M. Sheldrick, *Acta Crystallogr. A* 2008, **64**, 112. (b) G. M. Sheldrick, *Acta Crystallogr. C* 2015, **71**, 3.

S7. N. E. Brese and M. O'Keeffe, *Acta Crystallogr. B* 1991, **47**, 192.
